# Photofabrication of Highly Transparent Platinum Counter Electrodes at Ambient Temperature for Bifacial Dye Sensitized Solar Cells

**DOI:** 10.1038/s41598-018-31040-1

**Published:** 2018-08-27

**Authors:** Idris K. Popoola, Mohammed A. Gondal, Jwaher M. AlGhamdi, Talal F. Qahtan

**Affiliations:** 1 Laser Research Group, Physics Department & Center of Excellence in Nanotechnology, King Fahd University of Petroleum and Minerals, P.O. Box 5047, Dhahran, 31261 Saudi Arabia; 2 Department of Chemistry, College of Science, Imam Abdulrahman Bin Faisal University, Dammam, 31113 Saudi Arabia

## Abstract

Platinum (Pt) counter electrodes (CEs) have consistently shown excellent electrocatalytic performance and holds the record of the highest power conversion efficiency (PCE) for dye-sensitized solar cells (DSSCs). However, its use for large-scale production is limited either by high temperature required for thermal decomposition of its precursor or by wastage of the material leading to high cost or sophisticated equipment. Here, we report a novel photofabrication technique to fabricate highly transparent platinum counter electrodes by ultraviolet (UV) irradiation of platinic acid (H_2_PtCl_6_.6H_2_O) on rigid fluorine-doped tin oxide (FTO) and flexible indium-doped tin oxide (ITO) on polyethylene terephthalate (PET) substrates. The photofabrication technique is a facile and versatile method for the fabrication of Pt CEs for dye sensitized solar cells (DSSCs). The photofabricated Pt CEs were used to fabricate bifacial DSSCs with power conversion efficiencies (PCEs) attaining 7.29% for front illumination and 5.85% for rear illumination. The highest percentage ratio of the rear illumination efficiency to the front illumination efficiency (η_R_) of 85.92% was recorded while the least η_R_ is 77.91%.

## Introduction

Third generation solar cells such as dye-sensitized solar cells (DSSCs), quantum-dots sensitized solar cells (QDSSCs) and recently perovskite solar cells (PSCs) have generated enormous research interests as they are expected to ultimately rival and possibly replace silicon solar cells in the pursuit of renewable and clean energy utilizing the abundance energy of the sun. The research interests attracted by these third generation solar cells are due to their solution processing capabilities, low costs, easy fabrication techniques, efficient devices performances and potentials for application in flexible devices^1–4^. Since the work of Gratzel in 1991^5^, various components of DSSCs such as the substrates, photoanodes, sensitizers (dyes), electrolytes and the counter electrodes (CEs) have continued to be researched for new materials, synthesis methods and fabrication techniques. N-type photoanode with molecularly engineered dye has reached a record power conversion efficiency (PCE) of 13%^6^. TiO_2_ remains the most commonly used n-type semiconducting photoanodes^6–11^ ZnO, photoactive doped metal oxides and various nanocomposite materials have been reported as photoanodes in DSSCs^12–14^. Ruthenium (Ru) based sensitizers such as N3, N719 among others have become the *de facto* dye materials for DSSCs^15^. Liquid electrolytes are usually comprised of iodide/triiodide (I^−^/I^3−^) and cobalt complexes (Co^2+^/Co^3+^) redox couples^16,17^. Highly electrocatalytic materials like platinum (Pt) are used to reduced I^3−^ to I^−^ at the interface of the electrolyte/CE in order to sustain the flow of current and regenerate molecules of the oxidized sensitizer in the DSSCs devices^18,19^.

The CE is one of the foci interests of research toward the improvement and advancement of DSSCs. Various efforts have been on in utilizing other non-platinum electrocatalytic materials as well as developing new technique for the fabrication of Pt CE^1^. Some of the alternative materials that have been investigated and reported include polymeric conducting materials such as poly(3,4-ethylenedioxy-thiophene):poly(styrenesulfonate) (PEDOT: PSS)^1,20–23^, carbon materials such as carbon soot, graphene, carbon nanotube (CNT), carbon nanofiber (CNF) and graphite^24–27^, inorganic semiconducting chalcogenide compounds such as NiS, CoS, and CoSe^28^, platinic composite materials^29,30^, and other electrocatalytic composite materials^31,32^. Polymeric conducting materials and carbon materials have the advantages of low costs, solution processing and low temperature fabrication requirement. However, Pt has consistently shown excellent electrocatalytic performance and holds the record of the highest PCE for DSSCs^18^ Pt CEs are usually fabricated at an elevated temperature of 450 °C from platinic acid (H_2_PtCl_6_) precursor^33^ or vacuum sputtered from Pt target^34,35^. Thermal decomposition of H_2_PtCl_6_ for the fabrication of Pt CE is not suitable for material with lower thermal stability at the required elevated temperature for the synthesis of Pt. Hence, flexible Pt CE on conductive polyethylene naphtholate (PEN), polyethylene terephthalate (PET) and textile cannot be achieved through thermal decomposition process^18,19,36^. Sputtering deposition on the other hand results in wastage of material during deposition process, thereby, limiting its use for large scale production as it is not cost effective^18,19,36^.

Researchers have reported several attempts at fabricating Pt CEs at low temperature. Electrodeposition technique is one of such methods employed in the fabrication of Pt CEs at low temperature. This method which takes place at room temperature involves three electrodes configuration with transparent conductive oxide (TCO) substrate acting as the working electrode and electrolyte material containing platinic acid solution^37–39^. A cyclic voltammetry process is then performed using an electrochemical system. Electrophoretic deposition was used by Yin *et al*.^40^. They prepared H_2_PtCl_6_ glycol solution and preheated it under stirring for 6 h in an atmosphere of argon. ITO-PEN substrates were immersed in the resulting Pt-colloid and driven by a D.C. field of 1.6 Vcm^−1^. The Pt coated electrode was washed with deionized (DI) water and ethanol before being post thermally treated at 60 °C for 30 mins. Both electrodeposition and electrophoretic deposition methods have the shortcoming of large Pt loading in the electrochemical baths making them unfeasible for commercial production.

Some other alternative methods have however been reported. Chemical wet-chemistry reduction has been utilized for the fabrication of Pt CEs from H_2_PtCl_6_, employing acidic reducing agents without subsequent treatment. Matoh *et al*.^41^ employing chemical reduction method prepared H_2_PtCl_6_ in ethanol for the synthesis of nanostructured metallic Pt. The ethanolic Pt precursor was either spin-coated or drop coated on fluorine doped tin oxide (FTO) glass electrodes or indium doped tin oxide (ITO) PET flexible substrates and dried at room temperature. The coated surfaces were then treated with gaseous formic acid reducing agent at temperature of 100 °C for a period of 15 minutes. Hseih *et al*.^19^ used modified chemical reduction method to fabricate Pt CEs. Polyvinylpyrrolidone (PVP) served as surfactant, NaHBr_4_ as reducing agent, NaOH was used to achieve neutral platinic precursor and UV-ozone treatment was used to decompose the surfactant after deposition on FTO or ITO-PEN. Polyol reduction technique is a facile method of synthesis of Pt from H_2_PtCl_6_ whereby ethylene glycol (EG) is used as reducing agent. Mei *et al*.^42^ fabricated Pt CEs using EG solution of H_2_PtCl_6_.6H_2_O. The deposited precursor was thermally treated at 180 °C. The synthesized Pt on the substrates exhibited dense and porous Pt structures. The earlier resulting from growth of Pt on the substrates following the reduction while the latter is due to Pt nanoparticle precipitation. Li *et al*.^18^ used similar polyol method with modification of the pH of the H_2_PtCl_6_ and preheating the precursor solution at 110 °C for 30 mins. They as well pretreated the substrates with ‘piranha’ and 3-mercaptopropyl(trimethoxysilane) (MPTMS) to produce a thiol-functionalized silane self- assembled monolayer (SAM) film on the conductive substrates. The as-prepared functionalized substrates were soaked in the platinic EG solution for 12 h and rinsed with ethanol to eliminate undesirable residues and dried in nitrogen environment.

In this study, we photofabricated Pt CEs employing different solvents. Our photofabrication process utilized UV irradiation of deposited solutions of H_2_PtCl_6_ to achieve Pt CEs. This novel method of fabricating Pt CEs requires no pre/post-thermal treatment and was carried out in ambient environment. This method utilized minimal Pt loading. It requires no addition of surfactant which is required to be removed either by heating or other methods. It is as well properly suited for plastic substrates as no acidic treatment is performed in the fabrication process. Moreover, the high transmittances recorded for the photofabricated Pt CEs make them suitable for use in bifacial DSSCs. Bifacial DSSCs can be deployed as building windows and integrated electronic devices^43^.

## Results and Discussion

**Figure 1:**
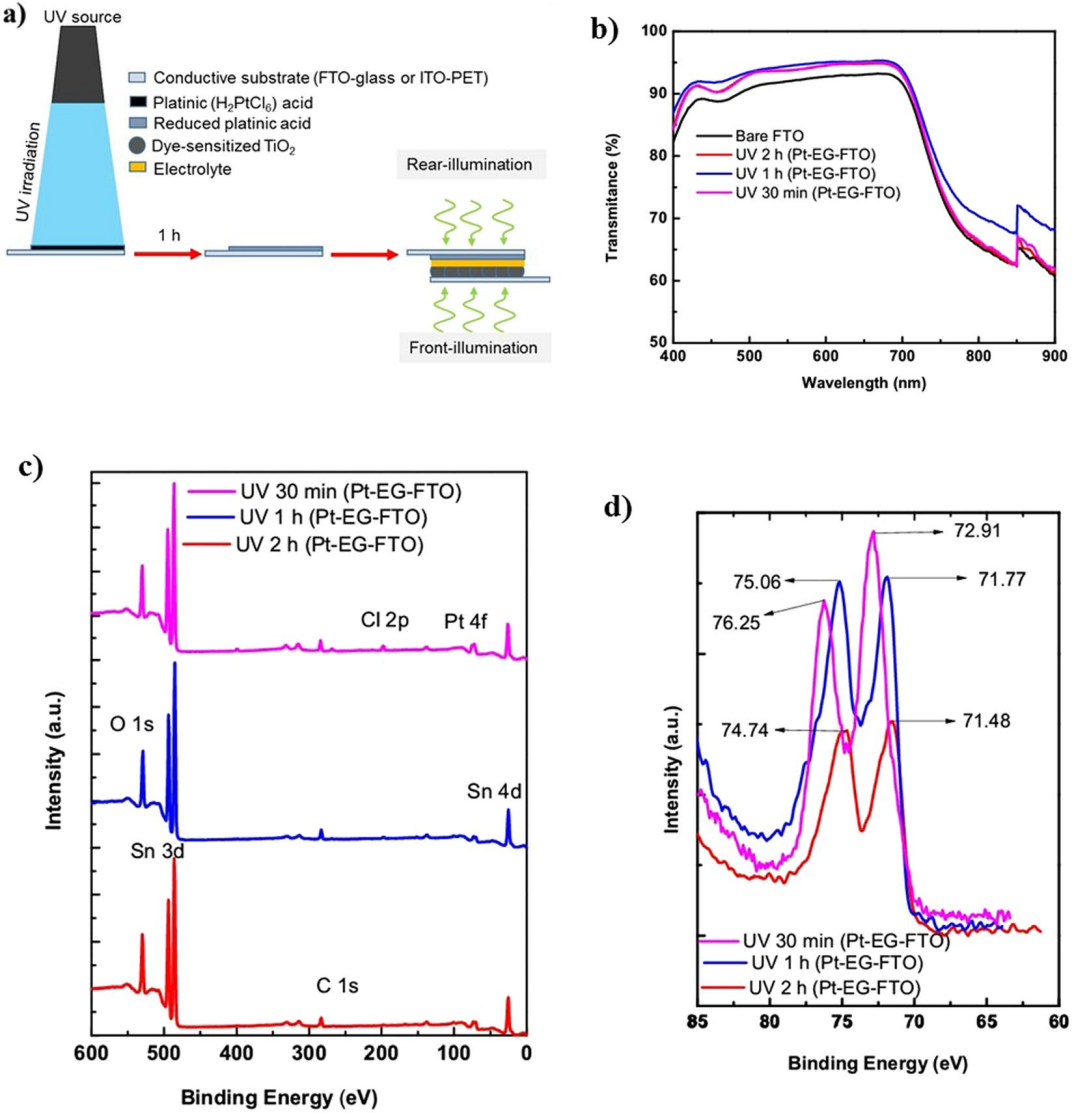
(**a**) Schematic showing photofabriation process and assembled DSSC (with front and rear illumination). (**b**) Transmittance spectra of photofabricated Pt CEs (Pt-EG-FTO) with different UV irradiation times and bare FTO for comparison. (**c**) XPS survey spectra of (Pt-EG-FTO) at different irradiation time. (**d**) XPS spectra comparing Pt 4 f peaks of the photofabricated Pt CE (Pt-EG-FTO) at different irradiation times.

Platinum counter electrodes were photofabricated under different fabrication parameters and characterized. Platinic acid precursor solution in EG making 0.02 M were spin-coated at 2000 rpm for 30 s at a ramp rate of 500 rpm. 20 ul of the EG solution of H_2_PtCl_6_ were deposited on an area of 0.25 cm^2^ before spinning. Three successive spin-coating cycles were conducted to ensure proper adhesion of the Pt precursor solution on the FTO substrates at the chosen spin-coating speed. After the spin-coating cycles have been completed, scotch tape used for the exposure of the coated area was removed before treatment with UV irradiation at ambient room conditions. On completion of the UV irradiation, the Pt precursor was reduced to Pt on the FTO substrate (Fig. 1a). The conversion was confirmed by different characterizations reported in this work. To get proper insight into the UV conversion process and attaining optimized parameters for the photofabrication process, effect of irradiation time was studied. The optimized loading amount of the Pt precursor was also investigated by three different spin-coating cycles. Finally, we examined the versatility of the photofabrication techniques by using different solvent than EG (in this case ethanol), drop coating method and flexible substrate (PET-ITO) was used in this work.

### Effect of Irradiation Time

To study the effects of irradiation time, samples were treated with different UV irradiation times of 2 h, 1 h and 30 min. The prepared samples with different irradiation times were characterized and used as CEs in fabrication of DSSCs. Figure 1a shows the transmittance spectra of the samples at different irradiation times and that of bare FTO glass. The transmittances of all the three samples at different irradiation times are higher than that of bare FTO glass within the visible light spectrum. For an understanding of these recorded enhancement of transmittance in the three samples, we examine the effect of UV irradiation on bare FTO glass. Figure S1a (Supporting Information) shows the transmittance spectra of a precleaned FTO glass that was treated with UV irradiation for different time intervals. The transmittance spectra were measured successively at 30 min, 1 h and 2 h irradiation times. We noticed the dependence of transmittance spectra on irradiation time. With this observation, the effect of UV irradiation on the resistivity of the FTO sample was investigated by measuring the resistance between two pre-marked points at 15 min, 30 min, 1 h and 2 h UV irradiation time. The resistance of the UV treated FTO was found to decrease and has dependency on the irradiation time as shown in Fig. S1b. Meanwhile, transmittance spectra of Fig. 1a suggest that the UV interaction with the Pt precursor on the FTO glass and subsequent formation of Pt metal equally played part in the enhancement of the transparency of the Pt-EG-FTO CEs within the visible light region. The sample that underwent 1 h UV irradiation photofabrication showed the highest transmittance across the visible light region of 400 nm to 720 nm. While those that were treated for 30 min and 2 h respectively had almost same transmittance spectra across same wavelengths.

The photoreduction of the H_2_PtCl_6_.6H_2_O in EG to Pt metal for the photofabricated CEs was investigated by XPS. The XPS spectra of the three samples with different UV irradiation times were compared to understand the effect of UV irradiation time on the photoreduction process. Figure 1b shows the XPS survey spectra of the three different samples with 3 cycles of spin-coating and UV irradiated for 30 min, 1 h and 2 h respectively. All three samples exhibit platinum peak at Pt 4 f orbital. Small Chlorine peak at Cl 2p orbital is observed to reduce with increase in UV irradiation time. At 1 h UV irradiation time the Cl 2p peak can be seen to have greatly reduced compare to UV irradiation time of 30 min. While the peak is absent at 2 h UV irradiation time. Figure 1c compares the platinum peaks of the three samples at the respective UV irradiation time. This might be due to degradation of the Platinum coating on the FTO. The Pt peak of UV irradiation of 30 min exhibit a binding energy of the Pt 4f_7/2_ at 72.91 eV shifted away from the 71.77 eV and 71.48 eV for the 1 h and 2 h UV irradiation times respectively. The Pt 4f_7/2_ binding energies of the of the 1 h and 2 h UV irradiation times are closest to the atomic platinum binding energy of 71.2 eV. The Pt peak at 2 h is seen to be lower intensity peak compared with that of UV irradiation time of 30 min and 1 h. Hence, optimized UV irradiation time for the photofabrication process is important.

**Figure 2:**
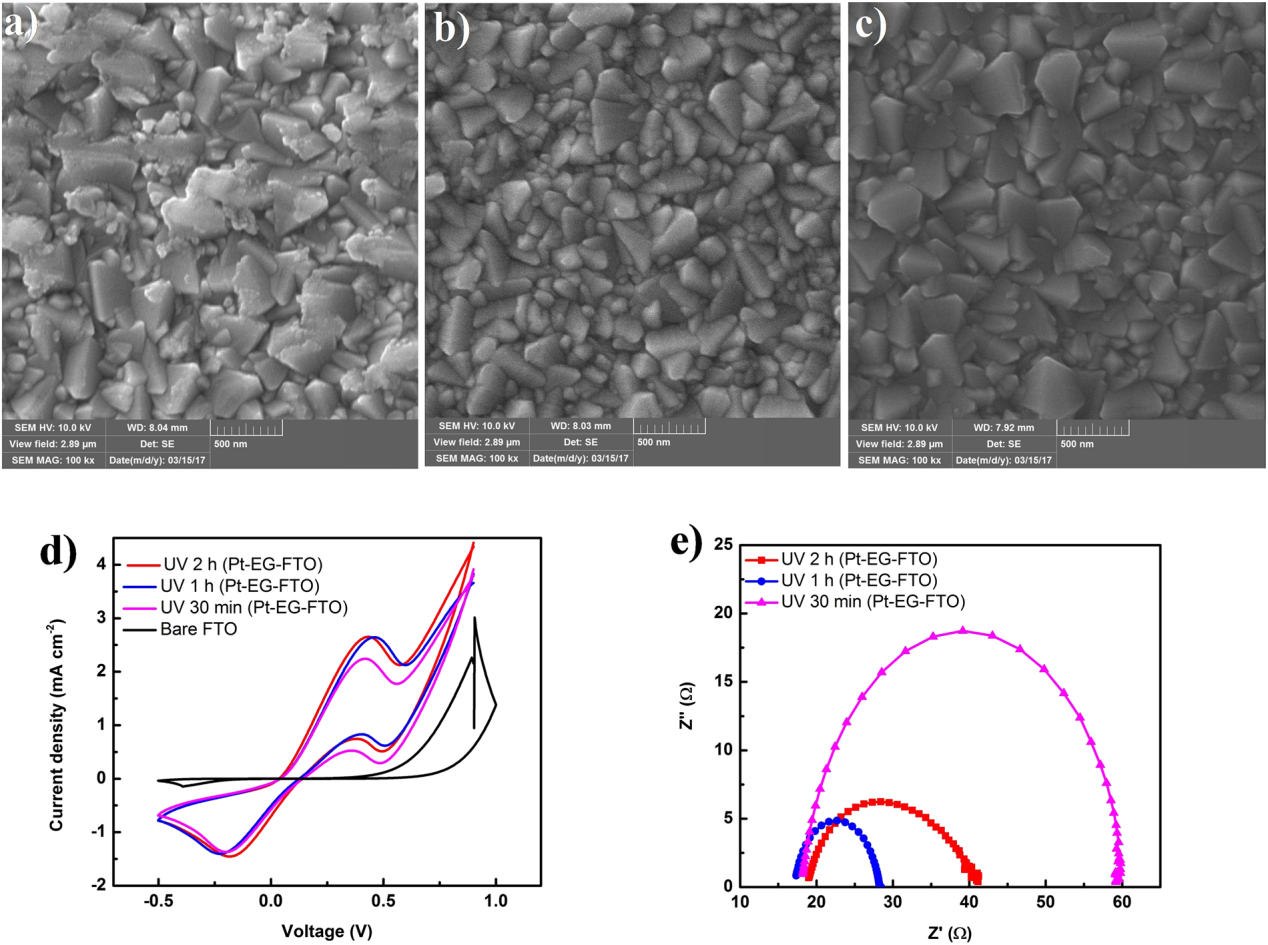
(**a**) SEM images of photofabricated Pt CE with 2 h UV irradiation time. (**b**) SEM images of photofabricated Pt CE with 1 h UV irradiation time. (**c**) SEM images of photofabricated Pt CE with 30 min UV irradiation time. (**d**) CV scan measurement of photofabricated Pt CEs (Pt-EG-FTO) at different irradiation times of 2 h, 1 h and 30 min. (**e**) Nyquist plots of photofabricated Pt CEs (Pt-EG-FTO) at different irradiation times of 2 h, 1 h and 30 min.

The SEM images shown in Fig. 2(a–c) for the three photofabricated Pt CEs with different UV irradiation times of 2 h, 1 h and 30 min indicate that Pt nanoparticles are well dispersed on the FTOs and no agglomerated site can be seen on the morphology of the photofabricated Pt CEs.

The electrochemical characterization of the samples was carried out to study the catalytic activity of the samples in triiodide/iodide electrolyte. Figure 2d shows the cyclic voltammograms of the samples. The bare FTO sample shows no catalytic activity as no reduction or oxidation is present in the CV scan of the sample. UV irradiated samples with 1 h and 2 h show both reduction and oxidation peaks that are aligned throughout the CV scan while sample with 30 min UV irradiation time exhibit a slight shift from them. CV measurement is particularly useful in understanding the regeneration of dye molecules from the triiodide/iodide electrolyte after the photoreduction of the dye molecule in generation of electron into the TiO_2_ photoanode material, as redox equilibrium is desired for the continuous functioning of the solar cells. The redox reaction at the electrolyte/photofabricated Pt CE interface is as given in equation (1):

1 $$\begin{array}{c}{{{\rm{I}}}_{{\rm{3}}}}^{-}+{{\rm{2e}}}^{-}\to {{\rm{3I}}}^{-}\\ {{\rm{3I}}}^{-}\to {{{\rm{I}}}_{{\rm{3}}}}^{-}+{{\rm{2e}}}^{-}\end{array}$$

The Nyquist impedance plot shown in Fig. 2e illustrate the charge transfer mechanism between the electrolyte and photofabricated Pt CEs in symmetric dummy cells. The fitting of the Nyquist plots is carried out within the NOVA 2.1 software. The equivalent circuit used in fitting the Nyquist plot is as shown in Fig. S2. The series resistance R_S_, charge transfer resistance R_CT_, constant phase element (CPE) and the exchange current density (J_0_) of the dummy cells are summarized in Table 1. The sample with 1 h UV irradiation exhibits the least series resistance and charge transfer resistance of 17.641 Ω and 10.639 Ω respectively. 2 h UV irradiated sample also performed better than 30 min UV irradiated sample. The surface area of the photofabricated Pt CEs as given by the CPE shows dependency with UV irradiation times (Table 1). The values of J_0_ are obtained from equation (2):

2 $${{\rm{J}}}_{{\rm{0}}}=({\rm{R}}\ast {\rm{T}})/({\rm{n}}\ast {\rm{F}}\ast {{\rm{R}}}_{{\rm{CT}}})$$

where R represents the molar gas constant, T ( = 298 K) is the absolute temperature, n represents the number of electrons involved in the triiodide reduction at the electrode/electrolyte interface having a value of 2 and F is the Faraday’s constant^33^. The Tafel plots for these Pt CEs are as shown in Fig. S3a. Pt-EG-FTO UV irradiated for 1 h exhibited the highest value for both the anodic and cathodic current densities.

**Table 1:** Nyquist impedance parameters of photofabricated Pt CEs.

Photofabricated Pt-CEs	R_S_ (Ω)	R_CT_ (Ω)	CPE (μF)	J_0_ (mA cm^−2^)
UV 2 h (Pt-EG-FTO)	18.21	22.697	11.91	0.57
UV 1 h (Pt-EG-FTO)	17.64	10.639	7.09	1.20
UV 30 min (Pt-EG-FTO)	18.17	40.788	5.69	0.32
UV 1 h (Pt-EtOH-FTO)	30.36	24.098	10.38	0.53
UV 30 min (Pt-EtOH-FTO)	37.65	143.53	9.20	0.09
UV 15 min (Pt-EtOH-FTO)	22.17	241.04	5.58	0.05
UV 1 h (Pt-EtOH-PET-ITO)	366.10	1655.30	0.39	0.008

From the transmittance, XPS and electrochemical characterizations results discussed above, we conclude that 30 min irradiation was not sufficient to reduce the Pt precursor to Pt metal. On the other hand, excessive UV exposure appears to be detrimental to the photofabrication process as is the case for the 2 h UV irradiated sample. Hence, 1 h irradiation time seems the optimal value for the UV photofabrication Pt CE technique.

### Effect of Solvents

**Figure 3:**
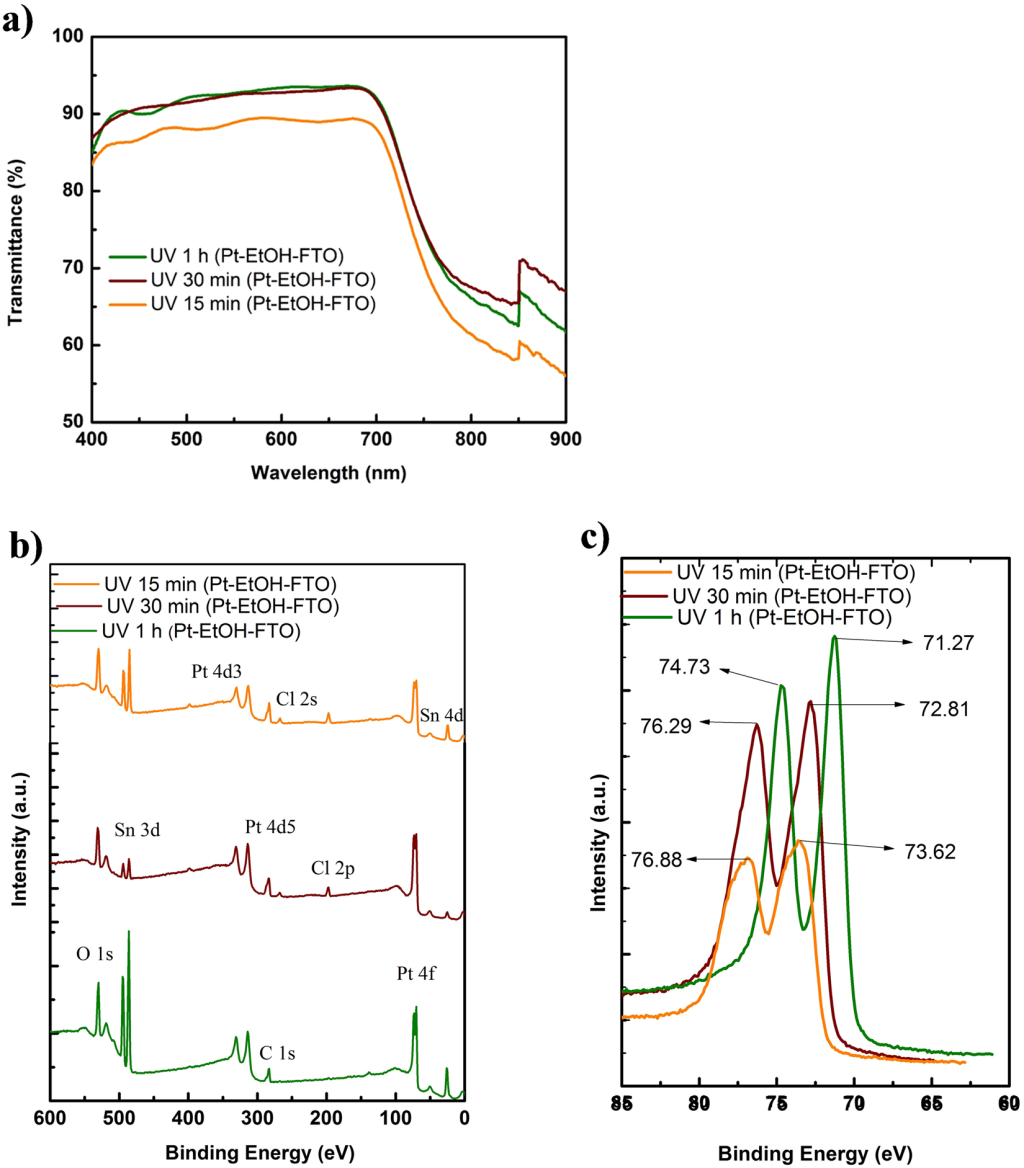
(**a**) Transmittance spectra of Pt CEs (Pt-EtOH-FTO) with different irradiation times. (**b**) XPS survey spectra of photofabricated Pt CEs (Pt-EtOH-FTO) with different UV irradiation time and (**c**) XPS spectra comparing Pt 4f peaks of photofabricated Pt CEs (Pt-EtOH-FTO) with different irradiation times.

The versatility of our photofabrication technique with respect to different solvents is reported. Here, we chose ethanol as a representative solvent of other suitable solvents that are used in the synthesis of Pt metal from H_2_PtCl_6_.6H_2_O precursor. Ethanol being a nontoxic solvent has an advantage of low boiling point of 78 °C over EG, it can therefore evaporate easily compared to EG. Owing to this advantage, drop casting method was used in depositing the platinic acid in ethanol precursor for the photofabrication process. The drop casting method utilized much lower platinic acid solution, leading to minimal Pt loading as compared to spin-coating process that results in wastage of material. For the drop casting process, 30 ul of 0.02 M ethanol solution of H_2_PtCl_6_.6H_2_O was dropped on an exposed area of 0.25 cm^2^ of FTO glass. Three samples were prepared using this approach and UV irradiated for 1 h, 30 min and 15 min, respectively. Figure 3a shows the transmittance spectra of the photofabricated Pt CEs from the ethanolic platinic acid solution. All the transmission spectra of the three samples are greater than the transmittance spectrum of bare FTO across the visible light wavelength region.

Figure 3b is the XPS spectra of the photofabricated Pt CEs from ethanol based platinic acid solution. All three samples at different UV irradiation time show much prominent platinum peaks than platinum peaks of all samples photofabricated from EG platinic acid solution. This indicates that there are better and more efficient platinum loading for drop coated precursor samples than there are for the spin-coated samples. The effect of UV irradiation time can as well be seen from the XPS spectra. As UV irradiation time increases from 15 min to 1 h, the platinum peaks can be seen to increase with respect to the irradiation time (Fig. 3c). Meanwhile, the Chlorine peaks decrease with increase in UV irradiation time (Fig. 3b), confirming the photoreduction of the H_2_PtCl_6_.6H_2_O in ethanol to Pt. Pt-EtOH-FTO with 1 h UV irradiation time exhibited Pt 4f_7/2_ peak at a binding energy of 71.27 eV, while Pt-EtOH-FTO with 30 min and 15 min UV irradiation had a shifted Pt 4f_7/2_ peaks at binding energies of 72.81 and 73.62 eV respectively.

**Figure 4:**
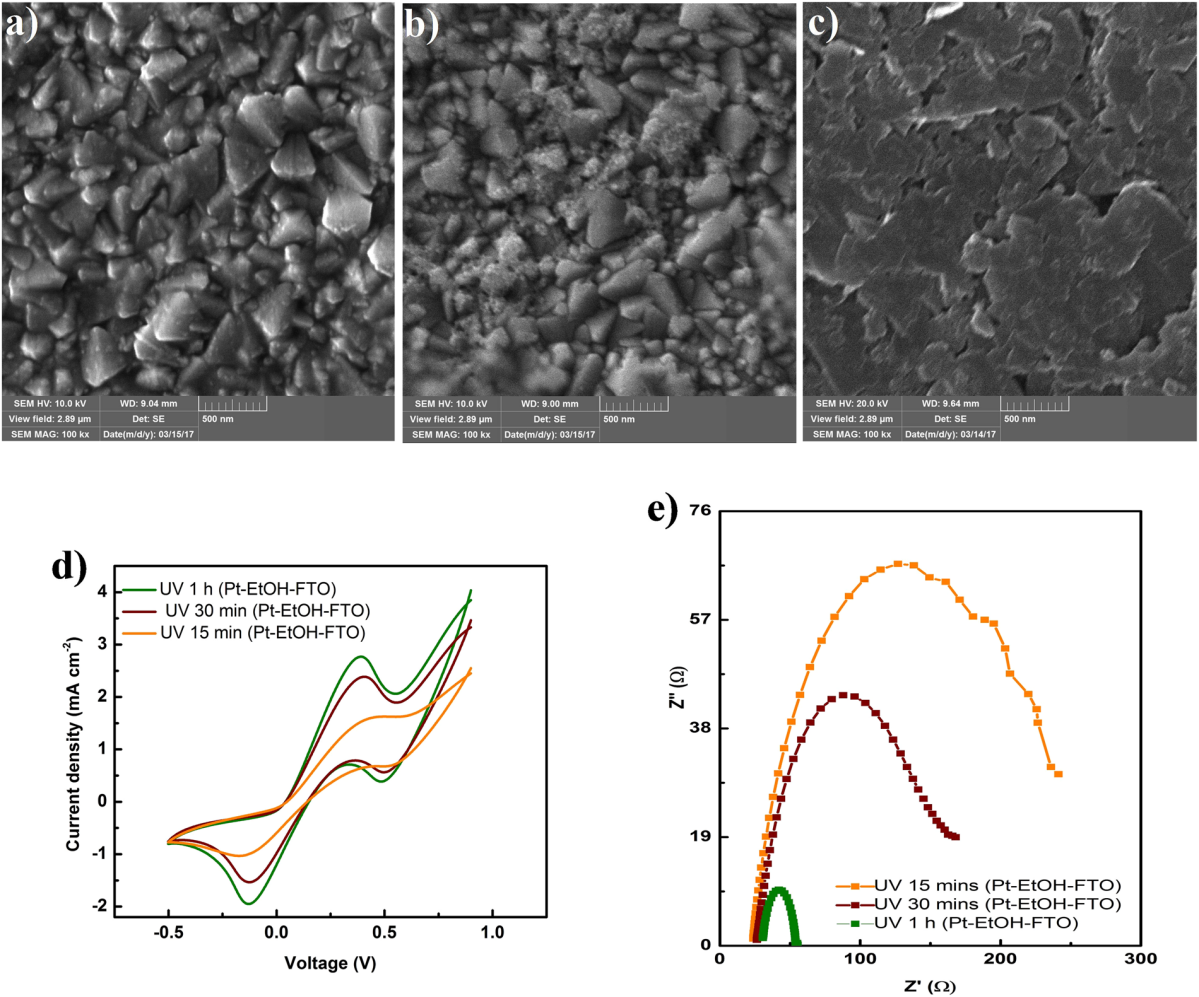
(**a**) SEM images of photofabricated Pt CE with 1 h UV irradiation time. (**b**) SEM images of photofabricated Pt CE with 30 min UV irradiation time. (**c**) SEM images of photofabricated Pt CE with 15 min UV irradiation time. (**d**) CV scan measurement of photofabricated Pt CEs (Pt-EtOH-FTO) at different irradiation time of 1 h 30 min and 15 min. (**e**) Nyquist plots of photofabricated Pt CEs (Pt-EtOH-FTO) at different irradiation time of 1 h, 30 min and 15 min.

The SEM images of photofabricated at different irradiation time are presented in Fig. 4. Figure 4a shows similarly well dispersed Pt particles on the FTO. However, Fig. 4c shows a different morphology of sheet and cloud-like structures indicating that the ethanolic platinic acid solution has only been partially photoreduced, While Fig. 4b shows traces of the sheet and cloud-like structures that are seen in Fig. 4c, further underscoring that 30 min UV irradiation was not sufficient for the photofabrication process.

The CV scan measurement of the photoreduced ethanolic platinic acid based Pt CEs are presented in Fig. 4d. Consistent with the XPS spectra and SEM images, 15 min UV irradiated sample showed poor catalytic activity as it exhibits little reduction and oxidation peaks in the CV scan measurement. On the other hand, 1 h UV irradiated sample showed a more prominent reduction and oxidation peaks, making it exhibits very good catalytic activity. Pt CE photofabricated with 30 min UV irradiation equally manifest good catalytic activity.

The Nyquist plot parameters of all ethanolic based photofabricated Pt CEs are presented in Table 1 and the plots are shown in Fig. 4e. Owing to the good Pt loading and high catalytic activity UV 1 h (Pt-EtOH-FTO) has a small series resistance and small charge transfer resistance. The Nyquist plot further confirmed the insufficiency of 15 irradiation time for the photofabrication process. The series and charge transfer resistances are to be higher than all photofabricated Pt CEs. CPE follows similar dependency with UV irradiation time as that of Pt-EG-FTO CEs. The calculated J_0_ values for the Pt-EtOH-FTO are as well listed in Table 1. Tafel plots in Fig. S3b shows Pt-EtOH-FTO with 1 h UV irradiation having the highest current density values for the anodic and cathodic current densities as compared with those of 30 and 15 min UV irradiation time.

### Photofabrication of Pt on PET-ITO

**Figure 5:**
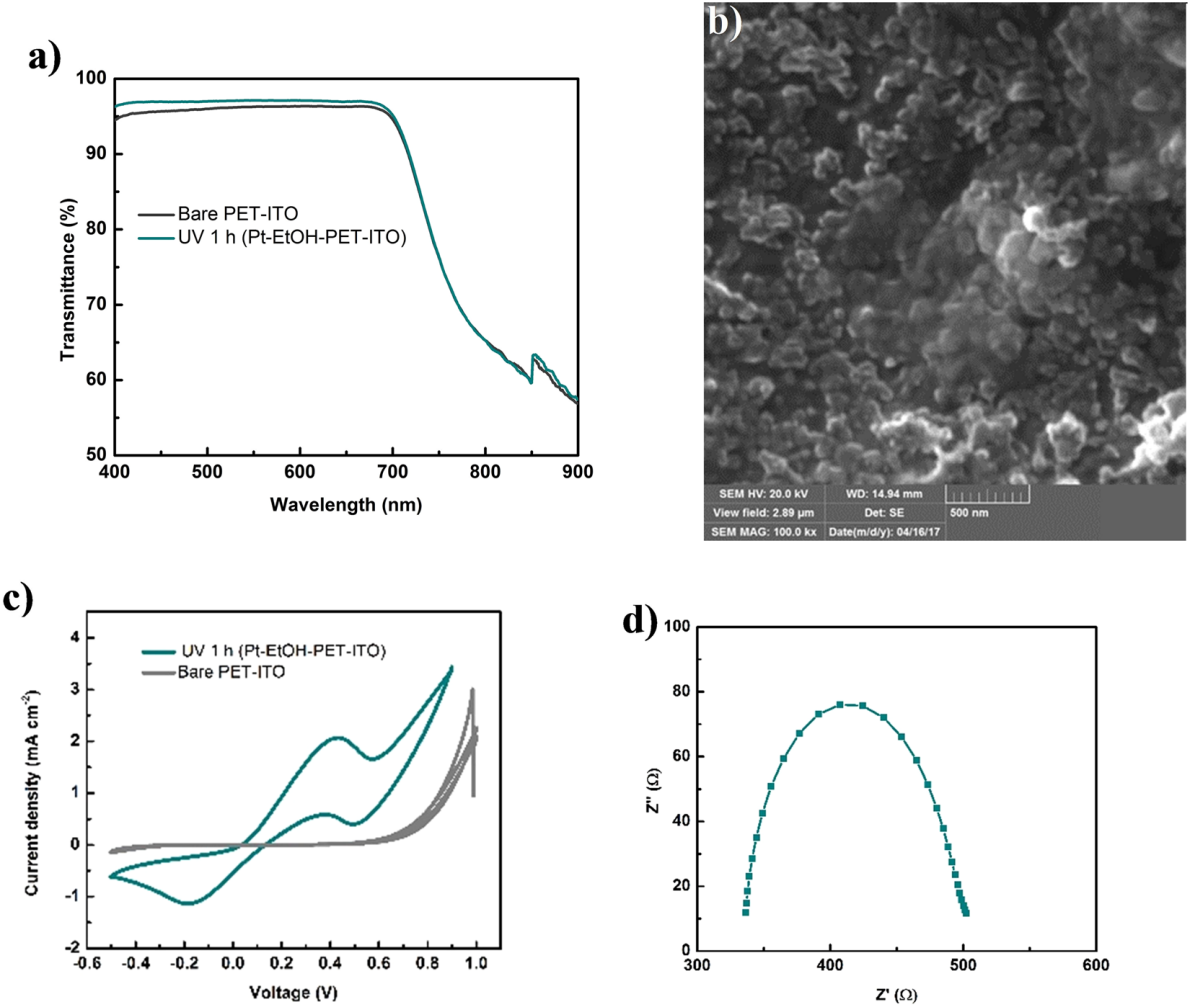
(**a**) Transmittance spectra of photofabricated Pt flexible CE and bare PET-ITO substrate. (**b**) SEM image of photofabricated Pt CE (Pt-EtOH-ITO-PET) with 1 h UV irradiation time. (**c**) CV scan measurement of photofabricated Pt CE (Pt-EtOH-PET-ITO) at irradiation time of 1 h as compared with bare PET-ITO substrate. (**d**) Nyquist plot of photofabricated Pt CE (Pt-EtOH-PET-ITO) at irradiation time of 1 h.

Flexible Pt CE on PET-ITO (Pt-EtOH-ITO-PET) was photofabricated as a demonstration of the versatility and potential area of application of the photofabrication technique. 5 ul ethanolic platinic acid precursor was drop casted on an exposed area of 0.25 cm^2^ of PET-ITO substrate and then treated with 1 h UV irradiation time. The ambient temperature of not more than 40 °C of the UV irradiation intensity particularly make it suitable for use on flexible substrates. Figure 5a is the transmittance spectra of the photofabricated Pt flexible CE. The obtained spectra show improvement of the transparency of the photofabrciated Pt flexible CE as compared to bare PET-ITO substrate. This is consistent with the transmittance results obtained for photofabricated Pt CEs on FTO substrates. Figure 5b shows the SEM morphology image of the Pt flexible CE with well dispersed Pt seen in the image and some agglomeration sites can as well be seen in the image. The catalytic activities of photofabricated Pt flexible CE were investigated by CV scan, EIS Nyquist measurement and Tafel plot. Figure 5c and d show the CV scan measurement and Nyquist plot of the photofabricated Pt flexible CE. The Tafel is as shown in Fig. S3c.

### Solar Cells Performance

**Figure 6:**
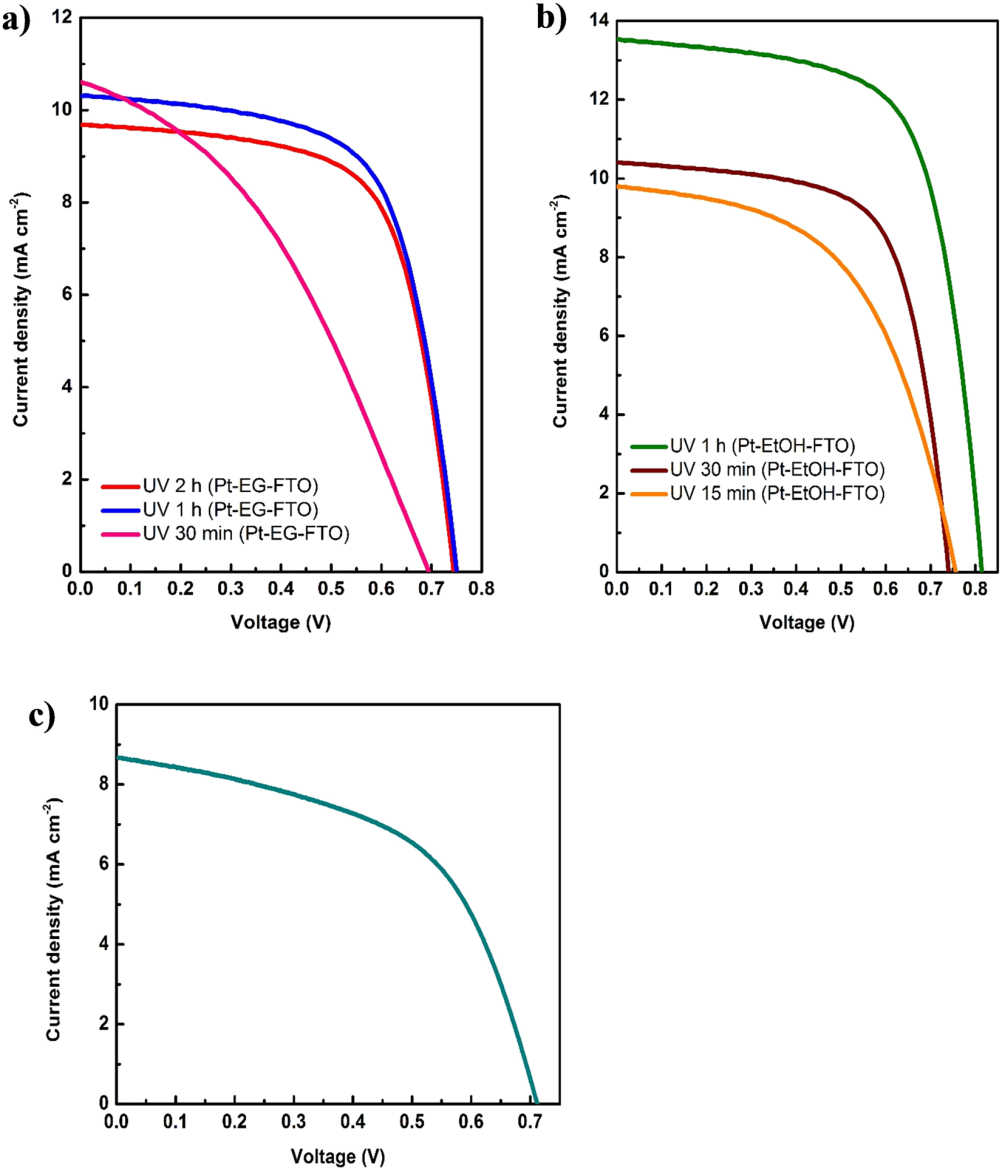
(**a**) I-V curve of DSSCs based on photofabricated Pt CEs (Pt-EG-FTO). (**b**) I-V characteristics curve of DSSCs with photofabricated Pt CEs (Pt-EtOH-FTO) and (**c**) I-V characteristic curve of flex-DSSC with photofabricated Pt flexible CE (Pt-EtOH-ITO-PET).

The photofabricated Pt CEs were used for the fabrication of bifacial DSSCs (Fig. 1a) and their photovoltaic performances were measured under the illumination of AM 1.5 G solar simulator at 100 mW cm^−2^ light intensity. Figure S4a shows typical DSSCs fabricated using the photofabricated Pt CEs while Fig. S4b shows the image of the fabricated flexible DSSC (flex-DSSC) utilizing the photofabricated Pt flexible CE. The front and rear illuminated DSSCs I-V parameters are summarized in Table 2. Figure 6a–c illustrate the front illumination I-V curve for the spin-coated (Pt-EG-FTO), ethanol based drop coated (Pt-EtOH-FTO) and the flexible (Pt-EtOH-ITO-PET) CEs. Figure S5(a–c) show the rear illumination I-V curves for the DSSCs fabricated with the respective Pt CEs. It is seen that the Pt CEs with 1 h UV irradiation time outperformed other Pt CEs with UV irradiation time other than 1 h for both EG and EtOH based fabricated Pt CEs respectively. Further confirming the good catalytic activities of Pt CEs fabricated with 1 h UV irradiation time as established by electrochemical CV, EIS and Tafel characterizations. Solvent effect shows EtOH based Pt CEs to be better and more efficient for DSSCs than EG based Pt CEs. Pt-EtOH-FTO CE with 1 h UV photoreduction exhibited the best performance efficiency of 7.29% and the highest open circuit voltage V_OC_ of 810 mV for front illumination. In a similar trend, DSSC utilizing Pt-EtOH-FTO CE with 30 min UV irradiation time performed better than all DSSCs fabricated with Pt-EG-FTO CEs with efficiency of 5.07% as compared to the best EG based CEs of 5.01% (for UV 1 h (Pt-EG-FTO)). Flexible DSSC based on UV 1 h (Pt-EtOH-ITO-PET) CE and employing ZnO as photoanode recorded a PCE of 3.26%.

**Table 2:** IV characteristics parameters of photofabricated Pt CEs DSSCs.

Photofabricated Pt-CEs	Illumination	J_SC_ (mA cm^−2^)	V_OC_ (V)	FF (%)	η (%)	η_Ρ_ (%)
UV 2 h (Pt-EG-FTO)	Front	9.68	0.74	66.47	4.76	85.92
	Rear	8.32	0.74	66.43	4.09	
UV 1 h (Pt-EG-FTO)	Front	10.31	0.75	64.9	5.01	85.42
	Rear	8.82	0.75	65.15	4.28	
UV 30 min (Pt-EG-FTO)	Front	10.59	0.69	38.49	2.81	79.36
	Rear	8.57	0.67	38.88	2.23	
UV 1 h (Pt-EtOH-FTO)	Front	13.53	0.81	66.56	7.29	80.25
	Rear	10.89	0.802	67.03	5.85	
UV 30 min (Pt-EtOH-FTO)	Front	10.4	0.74	66.77	5.07	77.91
	Rear	8.16	0.72	67.18	3.95	
UV 15 min (Pt-EtOH-FTO)	Front	9.79	0.75	53.1	3.92	78.57
	Rear	7.93	0.74	52.62	3.08	
UV 1 h (Pt-EtOH-PET-ITO)	Front	8.67	0.71	53.35	3.26	79.75
	Rear	7.14	0.687	53.02	2.6	
Pt-EtOH-FTO @ 450 °C	Front	15.49	0.84	57.73	7.54	35.94
	Rear	7.225	0.759	49.51	2.71	

*η_R_ is the percentage ratio of the rear illumination to the front illumination.

All DSSCs employing photofabricated Pt CEs retained more than 77% of their front illumination efficiencies when illuminated from the rear. The percentage ratio of the rear illumination efficiency to the front illumination efficiency (η_R_) is given in Table 2. The η_R_ trend is consistent with the reported transmittance spectra of the photofabricated CEs. UV 2 h (Pt-EG-FTO) CE retained the highest percentage conversion efficiency ratio at 85.92% slightly above 85.42% of UV 1 h (Pt-EG-FTO) CE. Flexible DSSC recorded η_R_ of 79.75%. Hence, our photofabrication technique proved adequate for utilization in bifacial DSSCs. Difference in PCEs between front and rear illumination of DSSCs is observed to be largely due to the reduced photocurrent density of the rear illuminated DSSCs. This reduction in photocurrent density can be ascribed to electrolyte layer in the cell which behaved as a barrier between the incident light radiation and the dye sensitizer. The electrolyte is known to reflect incident light away, thereby reducing the amount of light available for the photoexcitation of the dye molecules.

Figure S6a shows pictures of typical transparent photofabricated Pt CEs (upper left – UV 15 min (Pt-EtOH-FTO), upper right – UV 30 min (Pt-EtOH-FTO), and bottom left – UV 1 h (Pt-EtOH-FTO)) and opaque thermally fabricated Pt CE (bottom right). As a reference for comparison, thermally fabricated Pt CE at 450 °C was used to fabricate DSSC. An efficiency of 7.54% was recorded slightly above the best photofabricated Pt CE DSSC. The photovoltaic parameters for this cell are listed in Table 2 for both front and rear illumination. Figure S6c showed the front and rear illumination I-V curves for the DSSC fabricated with thermally prepared Pt CE. The rear illumination photovoltaic performance significantly deviates from the performance recorded for the front illumination. The deviation can be seen to result from the drastic drop in photocurrent density of the rear illuminated DSSC which consequently led to significant loss in fill factor. The high reflectance (low transmittance) of the thermally prepared Pt CE as shown in Figure S6b is responsible for the observed loss in photovoltaic parameters with a significantly reduced PCE of 2.71%. A η_R_ 35.94% was recorded for this cell, 41.97% less difference when compared with the photofabricated CE having the least η_R_ of 77.91%.

## Conclusion

We develop a novel photofabrication technique for the fabrication of highly transparent Pt CEs with the aid of UV irradiation on rigid FTO glass and flexible PET-ITO substrates. The facile and versatile photofabrication technique was used to fabricate Pt CEs that showed better transmittance across the visible light spectrum of 400 to 700 nm wavelengths than bare FTO glass and bare PET-ITO substrates. UV irradiation was found to improve both the transmittance and conductivity of bare FTO glass and improved transmittance of photofabricated Pt CEs was found to be a function of UV irradiation time. XPS spectra confirmed the photoreduction of H_2_PtCl_6_.6H_2_O to Pt metal CEs. XPS results established 1 h UV irradiation as the optimal photofabrication time. Catalytic activities of the photofabricated Pt CEs studied by CV scan measurement, EIS and Tafel plot are found depend on UV irradiation time as complete photoreduction is necessary for a better catalytic performance of the photofabricated Pt CEs. SEM images revealed well dispersed Pt nanoparticles on both the FTO and PET-ITO substrates. Investigation of solvents effects showed that ethanol as a volatile liquid is more suitable for the photofabrication technique as minimal Pt precursor is used thereby helping to save cost as against EG based platinic acid precursor that required to be spin-coated on the substrate owing to it high boiling point that makes drop casting unsuitable for the photofabrication method which is carried out at maximum ambient temperature of 40 °C. Spin-coating deposition technique make use of more precursor material that is largely wasted making it cost ineffective. The photofabricated Pt CEs were used to fabricate bifacial DSSCs with PCEs attaining 7.29% for front illumination and 5.85% for rear illumination as compare with DSSC utilizing thermally fabricated Pt CE having PCE of 7.54% and 2. 71% for front and rear illumination respectively. The highest percentage ratio of the rear illumination efficiency to the front illumination efficiency (η_R_) of 85.92% was recorded while the least η_R_ is 77.91%.

## Materials and Method

### Materials

Platinic (H_2_PtCl_6_) acid, ethylene glycol (EG) purriss grade, FTO glass (7 Ωsq^−1^) and ITO-PET (14 Ωsq^−1^). Lithium perchlorate (LiClO_4_), iodine and lithium iodide (LiI) were purchased from Sigma Aldrich. Acetone, 2-propanol and methanol were purchased from Fisher scientific, Ti-Nanoxide T/SP paste, and N719 sensitizer were all products of Solaronix, Switzerland. Iodide/triodide in acetylnitrile (AN) electrolyte was purchased from Chemsolarism.

### Platinum CE photofabrication process

FTO and ITO-PET substrates were cleaned successively using detergent, deionized (DI) water, acetone and 2-propanol for 1 hour by ultrasonication process using Branson 3510. The substrates were heated at 70 °C for 20 min to completely remove the organic cleaning agents. 0.02 M H_2_PtCl_6_ solution in EG was prepared. The H_2_PtCl_6_ readily dissolved in the EG solvent at room temperature. 20 ul of the platinic acid solution was then spin-coated on the pre-cleaned FTO substrate with an exposed area of 0.25 cm^2^ at 2000 rpm for 45 s using Specialty Coating System (SCS) 6800 spin coater series. The H_2_PtCl_6_ solution coated FTO substrate was then exposed to UV irradiation using Lumen Dynamics Omnicure series 2000 at 2 W cm^−2^ at a distance of 5 cm from the platinic acid coated FTO substrate in an ambient environment for a specific duration of time. The UV light intensity temperature was measured by a homemade Arduino-based temperature sensor. The maximum temperature recorded at UV light intensity of 2 W cm^−2^ was 40 °C.

### **TiO**_**2**_**photoanodes fabrication**

TiO_2_ photoanodes were prepared by blade coating Ti-Nanoxide T/SP paste on an 0.25 cm^2^ exposed area of a pre-cleaned FTO glasses. The 0.25 cm^2^ exposed area was achieved by covering the FTO glass with scotch tape leaving only an area of 0.25 cm^2^ for coating of TiO_2_ photoanode. The TiO_2_ coated FTO was heated at 450 °C for 30 mins on a hot plate in open air. Prior to the heating, the masking scotch tapes were removed. After the TiO_2_ paste had been baked for 30 mins, the TiO_2_ photoanodes was allowed to cool down gradually to room temperature. The samples were then soaked in N719 dye solution for 24 h.

### ZnO flexible photoanode fabrication

For the flexible DSSC, ZnO semiconducting photoanode was employed due to its low temperature processing potential. ZnO dispersion in butanol was utilized for coating on ITO-PET. Prior to coating, the ZnO dispersion was stirred for 2 h at 60 °C to achieve needed viscosity for a paste-like ZnO, which was then blade coated on ITO-PET and sintered at 120 °C. The sample was allowed to cool to room temperature before being immersed in N719 dye solution for 24 h.

### N719 Dye Solution Preparation

N719 dye sensitizer was dissolved in methanol. The solution was sonicated for 30 min to dissolve the N719 dye. The prepared dye was used to sensitize all TiO_2_ photoanodes and ZnO flexible photoanode.

### Dye Sensitized Solar Cells Coupling

The DSSCs fabrication was completed by coupling the photoanodes and the photofabricated Pt CEs using acrylic super glue gel. Triiodide/iodide electrolyte was introduced into the cells before being hand-pressed to seal the electrolyte in between the electrodes and completing the cells fabrication. The cells were left for some minutes prior to measuring the IV characteristics.

### Characterization

XPS spectra of photofabricated CEs were carried out using Thermos-Scientific ESCALAB-250Xi System equipped with monochromatic Al Kα radiation (*hv* = 1486.6 eV). Spectra acquisition was done using a constant energy mode with pass energy of 100 and 30 eV for the survey and the narrow scans, respectively. The analysis chamber base pressure was 4 × 10^−10^ mbar. The photofabricated CE samples were mounted onto the sample holders with the aid of double-sided conductive adhesive tapes and outgassed in the sample loading chamber for 5 h at 2 × 10^−7^ mbar. The data acquisition was carried out using Thermo-Scientific Avantage software was used to acquire the XPS data.

The morphology of the fabricated samples was studied using Lyra TESCAN field emission scanning electron microscopy (FESEM) equipped with an accelerating voltage of 5 kV.

Transmittance spectra of the photofabricated samples were recorded using the Jasco 670 double beam spectrophotometer at wavelength range of 400 nm and 900 nm.

To study the catalytic activity of photofabricated Pt CE samples, three electrodes cyclic voltammetry measurement was conducted. Saturated calomel electrode (SCE) served as the reference electrode, platinum plate sheet electrode was used as the counter electrode while the photofabricated Pt electrodes were placed as the working electrodes in the setup. The electrolyte used contained 0.1 M lithium perchlorate (LiClO_4_), 0.01 M lithium iodide (LiI) and 0.001 M iodine (I_2_) all in acetonitrile (AN) solvent. The operating potential for the CV measurement ranges between −0.5 V to 1 V vs SCE. The CV measurement was carried out on Autolab PG302N equipped with NOVA 2.1 software.

The electrochemical impedance spectroscopy measurement of samples was carried using Autolab PG302N potentiostat equipped with NOVA 2.1 software. The Nyquist plot of the impedance parameters and tafel plots were carried out on the system. The operating frequency ranges from 0.1 Hz to 100 kHz at a voltage scan rate of 10 mV/s.

The I-V characteristics of the photovoltaic performance of the fabricated DSSCs utilizing photofabricated Pt CEs were measured using Autolab potentiostat PG302N equipped with NOVA 1.11 software. Oriel lamp solar simulator calibrated to 100 mW cm^−2^ was used as light illumination source for the I-V characteristic measurement. An area of 0.25 cm^−2^ was exposed for the measurement.

## Electronic supplementary material

Supplementary Information
