# Supplementary material for: Photofabrication of Highly Transparent Platinum Counter Electrodes at Ambient Temperature for Bifacial Dye Sensitized Solar Cells
Source: Sci Rep. 2018 Aug 27;8:12864. doi: 10.1038/s41598-018-31040-1 (PMC6110791; doi:10.1038/s41598-018-31040-1)
Supplement: Supplementary file 1 — Supplementary Information [file 41598_2018_31040_MOESM1_ESM.docx]

**Photofabrication of Highly Transparent Platinum Counter Electrodes at Ambient Temperature for Bifacial Dye Sensitized Solar Cells.**

Idris. K. Popoola^a^, Mohammed. A. Gondal^a*^, Jwaher M. AlGhamdi^b^, and Talal F. Qahtan^a^

**^a^** Laser Research Group, Physics Department, King Fahd University of Petroleum and Minerals, P.O. Box 5047, Dhahran 31261, Saudi Arabia.

^b^Department of Chemistry, College of Science, Imam Abdulrahman Bin Faisal University, Dammam 31113, Saudi Arabia

*Corresponding author. E-mail address: magondal@kfupm.edu.sa (M.A. Gondal), Telephone: +96613-8602351/8603274;

**SUPPLEMENTARY INFORMATION**

**
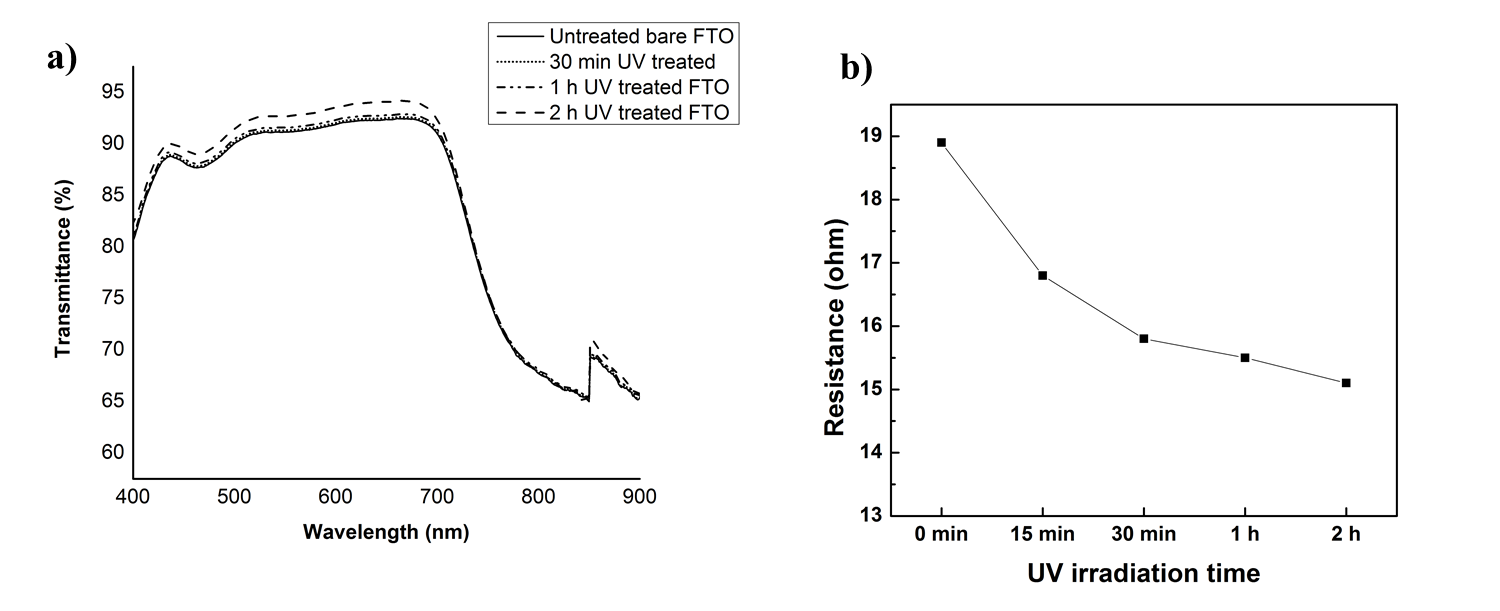
**

**Figure S1** a) Transmittance spectra of UV irradiated bare FTO glass at different irradiation time b) UV irradiation time versus resistance of bare FTO.


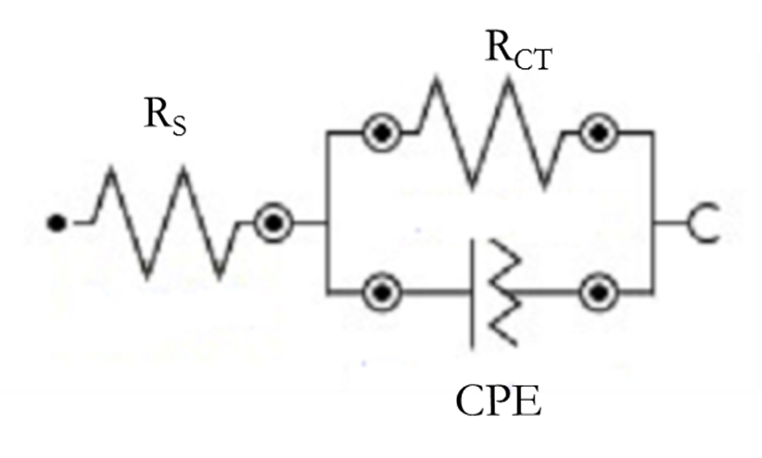


**Figure S2:** Equivalent circuit used for fitting the EIS Nyquist plots of photofabricated Pt CEs.

**Figure S3:** Tafel plots of a) Pt-EG-FTO CEs with UV irradiation time of 2 h, 1 h and 30 min b) Pt-EtOH-FTO CEs with UV irradiation time of 1 h, 30 min and 15 min and c) flexible Pt-EtOH-ITO-PET CE with 1 h UV irradiation time.

**Figure S4:** a) Photograph of a typical assembled cell samples using photofabricated Pt CEs on FTO substrates b) Flexible DSSC using photofabricated Pt CE on PET-ITO substrate.

**Figure S5:** I V curves of back illuminated DSSC a) DSSCs with Pt-EG-FTO CEs b) DSSCs with Pt-EtOH-FTO CEs and c) flex-DSSC with Pt-EtOH-ITO-PET CE.

**
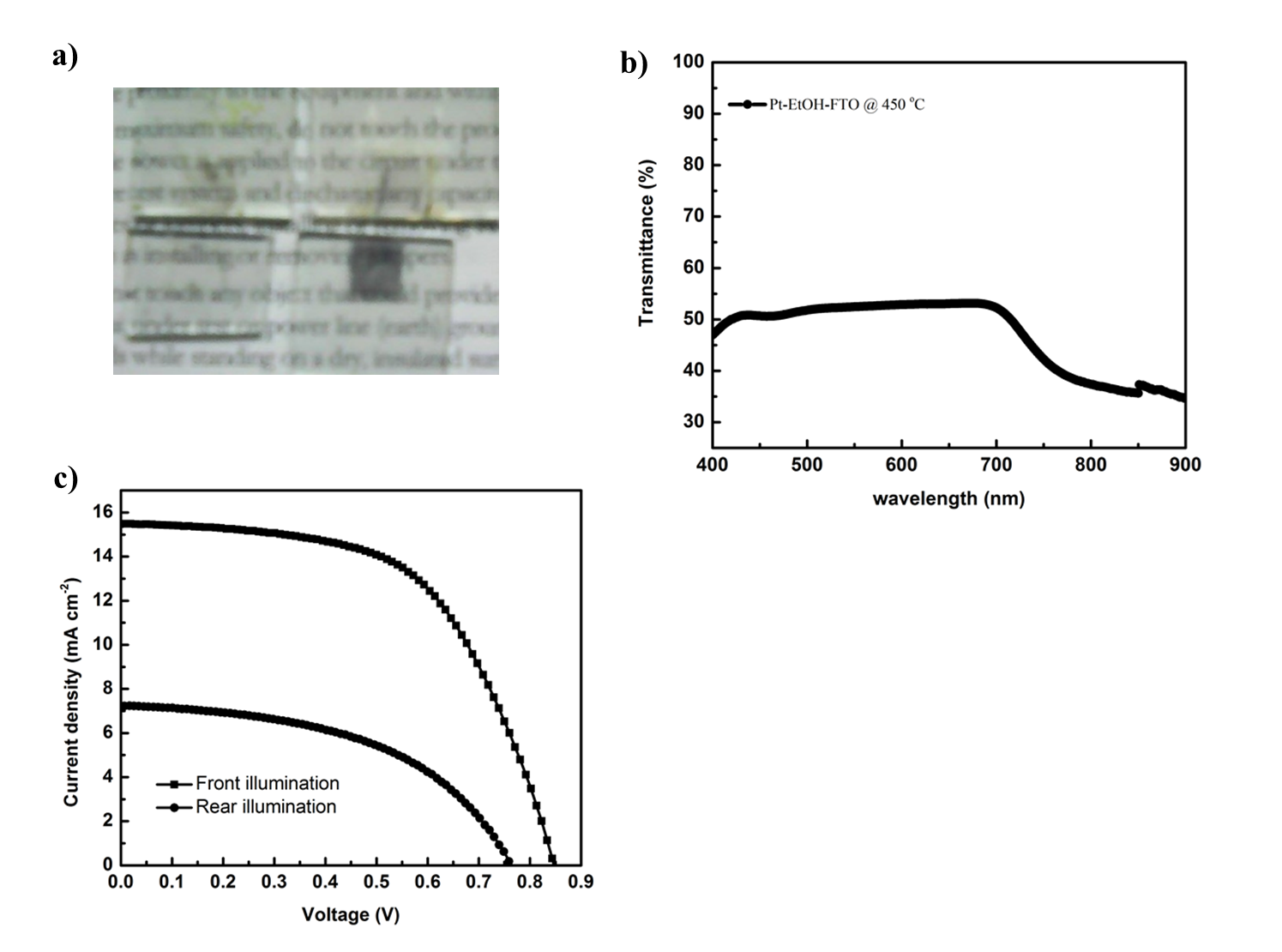
**

**Figure S6: :** a) Images of typical transparent photofabricated Pt CEs (upper left – UV 15 min (Pt-EtOH-FTO), upper right – UV 30 min (Pt-EtOH-FTO), and bottom left – UV 1 h (Pt-EtOH-FTO)) and opaque thermally fabricated Pt CE (bottom right) b) Transmittance spectra of thermally fabricated Pt CE @ 450 ^o^C c) IV curves of front and rear illuminated DSSC utilizing thermally fabricated Pt CE @ 450 ^o^C.
